# Combined Use of *C. butyricum* Sx-01 and *L. salivarius* C-1-3 Improves Intestinal Health and Reduces the Amount of Lipids in Serum via Modulation of Gut Microbiota in Mice

**DOI:** 10.3390/nu10070810

**Published:** 2018-06-24

**Authors:** Miao Long, Shuhua Yang, Peng Li, Xin Song, Jiawen Pan, Jianbin He, Yi Zhang, Rina Wu

**Affiliations:** 1 Key Laboratory of Zoonosis of Liaoning Province, College of Animal Science & Veterinary Medicine, Shenyang Agricultural University, Shenyang 110866, China; longmiao@syau.edu.cn (M.L.); yangshuhua0001@126.com (S.Y.); lipeng79625@163.com (P.L.); 13904227861@126.com (X.S.); panjiawen0101@163.com (J.P.); hejianbin69@163.com (J.H.); sihuo12345@sohu.com (Y.Z.); 2 College of Food Science, Shenyang Agricultural University, Shenyang 110866, China

**Keywords:** *Lactobacillus salivarius*, *Clostridium butyricum*, blood and biochemical indexes, intestinal microflora, intestinal morphology

## Abstract

The study was conducted to investigate whether combined use of *C. butyricum* Sx-01 and *L. salivarius* C-1-3 could improve the intestinal health and reduce the lipid levels in sera of mice and whether these benefits were related to regulating the intestinal microflora. Eighty Kunming male mice were divided into four groups with five replicates per group and four mice per replicate. Mice in the control group were administrated with 0.2 mL normal saline; mice in three experimental groups were daily orally administrated with 4 × 10^8^ cfu of *L. salivarius*, 4 × 10^8^ cfu of *C. butyricum*, and a combination thereof (2 × 10^8^ cfu of *L. salivarius*, and 2 × 10^8^ cfu of *C. butyricum*), respectively. The experiment lasted for 14 days. The results showed that the average daily feed intake (ADFI) and feed/gain (F/G) ratio of growing mice underwent no significant changes (*p* > 0.05); however, the average daily gain (ADG) tended to increase over short periods of time. The activities of SOD and GSH-Px in serum in the combination group were significantly increased (*p* < 0.05); The triglyceride, and total cholesterol, contents in serum in the combined treatment group were significantly decreased (*p* < 0.05); The total volatile fatty acids and butyric acid in faecal matter of mice in the experimental groups were all significantly increased at 14 days (*p* < 0.05); The length of villi, and the mucosal thickness of colon and caecum (*p* < 0.05) were significantly improved; The relative abundance of some bacteria with antioxidant capacity or decomposing cholesterol capacity or butyrate producing capacity was increased, while the relative abundance of some pathogenic bacteria was decreased in the colon. Furthermore, our results showed that the beneficial effects of the combined use of the two strains was higher than that of single use. Overall, the results demonstrated that the combined use of *C. butyricum* Sx-01 and *L. salivarius* C-1-3 can significantly improve intestinal health and reduce the amount of lipids in sera of mice. The reason for these effects might be that besides their own probiotic effects, combined use of the two strains could regulate the intestinal microflora.

## 1. Introduction

*Clostridium butyricum* (*C. butyricum*) is a Gram-positive anaerobe species, which can produce butyric-acid and survive at relatively high bile concentrations, lower pH, and temperature; it is often found in the gut of healthy humans and animals [1,2]. Most of strains of *C. butyricum* can produce butyrate, which has the ability to maintain gut immunological homeostasis [3,4] and can, therefore, help the proliferation of intestinal mucosal cells [5]. Certain strains of *C. butyricum* have often been hypothesised to have beneficial effects of prebiotics. Studies have demonstrated that some strains of *C. butyricum* can promote growth performance of broilers [6,7], balance the intestinal microflora of broiler chickens [3], and improve intestinal morphology [8]. Studies have found that *C. butyricum* MIYAIRI 588 can treat, and prevent diarrhoea induced by non-antimicrobial agents in rats [9], *C. Butyricum* CGMCC0313.1 has the ability to modulate colon homeostasis and the lipid profile in obese mice and can stimulate the immune system of mice [10,11], and a specific strain of *C. Butyricum* has the anti-oxidant and anti-apoptotic ability to exert neuroprotective effects against I/R injury in mice [12]. However, some studies also show that some strains of *C. butyricum* are related to botulism in infants or necrotizing enterocolitis [13].

*L. salivarius* species, which belongs to the *Lactobacillus genus*, has gained attention as a promising probiotic species. Certain strains of *L. salivarius* exhibit antibacterial and antifungal properties and have a good health effect on the intestine of mice, rats and humans for the alleviation of intestinal disease [14]. Specific strains of the species have been used to prevent and treat some chronic diseases, such as asthma, cancer and halitosis in humans and to reduce colonization by gastrointestinal (GI) pathogens in animal [15]. Studies showed that heat-killed *L. salivarius* could protect ftom the liver damage induced by alcohol in rats [16], *L. salivarius* LI01 exerted a good health-promoting effect in acute liver failure in humans [17]; *L. salivarius* MTC 1026 could inhibit the adhesion process of some pathogenic bacteria on Caco-2 cells in a competition assay [18]; *L. salivarius* B1 could elicit local immunomodulatory activities and increase the maturation of the intestinal mucosal immune system of piglets [19]. Although it seems that *L. salivarius* is safe for consumption by animals and humans. However, there is still a lack of information on the safety of many of the strains as prophylactics in animal and human health.

Although some stains of *C. butyricum* and *L. salivarius* have a beneficial effect on the health of the human and animal, it is necessary to carry out relevant tests to determine whether the strains for using as the probiotics is safe. Furthermore, the information is lacking on the effects of the combined use of the two strains on the intestine health. Therefore, we firstly studied the effect of *C. butyricum* sx01, *L. salivarius* C-1-3, which we had isolated from the health chicken and their combined use on blood and biochemical indices, volatile fatty acids, intestinal morphology in mice, and then studied their effect on intestine microflora. Through our research, we intended to reveal whether the two strains were safe for use and whether the effects of the combination use of them on the intestinal health of mice were related to their ability to modulate the intestinal microflora in mice.

## 2. Experimental Section

### 2.1. Probiotic Strains

Strains *C. butyricum* Sx-01 and *L. salivarius* C-1-3 [20] were isolated from the intestine of healthy chicken without being given medicine: they were identified by sequencing PCR-amplified 16S rDNA and physiological and biochemical characteristics and stored in our laboratory. The sequence information of the 16S rDNA gene were submitted to the GenBank, and their accession number was MH259843 and KP979479, respectively. The *C. butyricum* Sx-01 and *L. salivarius* C-1-3 were stored in broth supplemented with 30% (*v*/*v*) glycerol at −80 °C. The 1% (*v*/*v*) of *C. butyricum* Sx-01 or *L. salivarius* C-1-3 were cultured in Reinforced Clostridium Medium (RCM) broth and in de Man-Rogosa-Sharpe (MRS) broth at 37 °C for 24 h respectively before the experiment. The cells were collected after centrifugation (5000× *g*, 5 min) and washed three times with PBS solution. The cells were resuspended to the final concentration of 1 × 10^9^ viable bacterial cells per ml PBS. The suspensions were freshly prepared for each administration.

### 2.2. Animals and Treatment

Some 80 male Kunming mice (body mass 20 ± 2 g) were purchased from Liao Ning Chang Sheng Biotechnology Co., Ltd., Benxi, China. The mice were bred in a room at a temperature ranging from 22 to 24 °C and the mice were subjected to 12-h light/dark cycles and an atmosphere with a relative humidity of from 40 to 60%. Water and diet were provided ad labitum for the mice. The composition of the diets of the mice (%): corn flour 27%, bran 19%, rice 16%, bean cake 16%, fish powder 13%, calcium power 3%, bone powder 3%, yeast powder 2.3%, NaCl 0.5%, multivitamin 0.1%, trace elements 0.1%. The mice were acclimatised for one week after transportation. The experimental procedures have been approved by the Ethics Committee for Laboratory Animal Care (Animal Ethics Procedures and Guidelines of the People’s Republic of China) for the use of Shenyang Agricultural University, China. (Permit No. 264 SYXK<Liao>2011-0001).

Mice were randomly distributed into four groups, each group had five replicates, and each replicate set contained four mice. Animals within different treatment groups were treated daily by oral gavage (without anaesthesia) at 09:00 and 15:00 for 14 d as follows: Group A (control group) = administrated with 0.2 mL of normal saline; Group B (*L. salivarius* group) = administrated with 0.2 mL of *L. salivarius* (1 × 10^9^ cfu/mL); Group C (*C. butyricum* group) = administrated with 0.2 mL of *C. butyricum* (1 × 10^9^ cfu/mL); Group D (group of *C. butyricum* + *L. salivarius*) = administrated with 0.1 mL of *L. salivarius* (1 × 10^9^ cfu/mL) and *C. butyricum* (1 × 10^9^ cfu/mL), respectively. Upon intragastric administration on days 0, 5, 9, and 14, the mice mass, the mass gain, feed intake, and mortality were recorded.

### 2.3. Blood and Biochemical Indices

The mice were sacrificed, 24 h after final treatment, under anaesthesia. Afterwards, we collected the blood samples and separated the serum. Routine blood tests were run to assay the whole blood of mice in each group by using a haematology analyser (HBVet-5 model, Sinnowa, Nanjing, China).

In the experiment, the indices of antioxidation, the biochemical levels of the liver were tested by using commercial kits (Nanjing Jiancheng Bioengineering Institute, Nanjing, China). The details of all determination procedures followed the manufacturer’s instructions for the commercial kits (Nanjing Jiancheng Bioengineering Institute, Nanjing, China). SOD was detected by WST-1 method; GSH-ST, GSH-Px were detected by colorimetry method; MDA was detected by TBA method; TC was detected by single-agent GPO-PAP method; TP was measured by Coomassie brilliant blue method; GSH, GOT, GPT were detected by micro-enzyme method; ALB was determined by the bromocresol-chlorine method; and TG was detected by GOD-PAP method.

### 2.4. Volatile Fatty Acids Analysis

For the Volatile fatty acids (VFAs) analysis, the modified method was applied to determine the VFAs concentration in the faeces [21,22]. Firstly, 2–5 g faecal samples were taken and mixed with KCl solution (0.4 mol/L) in an ice-water bath for 2 min, then the supernatants were packed after the samples were centrifuged at 10,000 rpm for 10 min at 4 °C. The concentrations of VFAs were determined by using an Agilent gas chromatograph. The 5 mL of the supernatant was added to 2 mL of 25% metaphosphoric acid in 3 M sulfuric acid. Total VFAs and three individual VFAs (acetic acid, propionic acid, and butyric acid) were separated and quantified using gas chromatography (Agilent Technologie 7890B GC system, Santa Clara, CA, USA) using a packed glass column DM-FFAP (30 m × 250 μm × 250 μm, 40–250 °C). The external standard method was used for this test. Chromatographically pure grades of acetic acid, propionic acid, and butyric acid were used as mixed standards. Draw a standard curve and calculate the concentration of volatile fatty acids in faces

### 2.5. Tissue Sections

Before conducting routine processing and paraffin embedding, the sections of ileum, colon, and caecum were set in 10% formalin. According to the methods provided in the reference of Jeong et al. (2018) [23], we used hematoxylin and eosin to stain the testis sections and inspected them by using a photomicroscope. Las EZ image pre-processing software was used to measure various intestinal segments of the villus height (VH), width (VW), crypt depth (CD), and mucosal thickness, and then VH/CD was calculated. Data SAS 9.4 was used to analysis these data.

### 2.6. Colon Microbial Analysis

Fresh colon faecal samples from seven mice randomly selected from each group were collected during the final 14 days for colon microbial analysis.

To amplify V3-V4 region of 16S rRNA gene for Illumina deep sequencing, universal primers, 338F:5′-ACTCCTACGGGAGGCAGCA-3′ and 806R:5′-GGACTACHVGGGTWTCTAAT-3′, were used [24]. The PCR was performed in a total reaction volume of 20 µL: H_2_O 13.25 µL, 10 × PCR ExTaq Buffer 2.0 µL, DNA template (100 ng/mL) 0.5 µL, prime1 (10 mmol/L) 1.0 µL, primer (10 mmol/L) 1.0 µL, dNTP 2.0 µL, ExTaq (5 U/mL) 0.25 µL. After an initial denaturation at 95 °C for 5 min, an amplification was performed by 30 cycles of incubations for 30 s at 95 °C, 20 s at 58 °C, and 6 s at 72 °C, followed by a final extension at 72 °C for 7 min. Then the amplified products were purified and recovered using 1.0% agarose gel electrophoresis method. Finally, the library construction and sequencing steps were performed by Beijing Biomarker Technologies Co., Ltd., (Beijing, China).

### 2.7. Bioinformatics Analysis

The bioinformatic analysis in this study was completed at the Biomarker biocloud platform (www.biocloud.org). To obtain the raw tags, paired-end reads were merged by FLASH (v1.2.7, http://ccb.jhu.edu/software/FLASH/) [25]. Then raw tags were filtered and clustered in the next steps. The merged tags were compared to the primers, and the tags with more than six mismatches were discarded by FASTX-Toolkit. Tags with an average quality score <20 in a 50-bp sliding window were truncated using Trimmomatic (http://www.usadellab.org/cms/?page=trimmomatic) [26] and tags shorter than 300 bp were removed. We identified possible chimeras by employing UCHIME, a tool included in mothur (http://drive5.com/uchime). The denoised sequences were clustered using Qiime UCLUST module and tags with similarity ≥97% were regarded as an OTU. Taxonomy was assigned to all OTUs by searching against the Silva databases (Release119, http://www.arb-silva.de.) using the RDP classifier within QIIME. The statistical processing of all the data was undertaken using SPSS17.0 software (IBM, Almon, NY, USA). The one-way ANOVA were used to analyse differences between groups and LSD method was used for multiple comparisons. Results were presented as the mean ± standard error (X ± SE). Mean values were proven to be different at *p* < 0.05.

## 3. Results

### 3.1. Growth Performance

During the experiments, there were no mice that died or suffered from diarrhoea in any group. As shown in Table 1, the average daily gain and the average daily feed intake were not significantly different between these groups (*p* > 0.05), but the Feed/Gain Ratio in the treated groups showed a downward trend compared with that in the control group.

### 3.2. Blood Routine Indices

As shown in Table 2, compared with the control group, the MCV, MCHC, and RDW-SD were significantly different in all treated groups (*p* < 0.05), however, the other indices were not significantly different in all treated groups (*p* > 0.05). These results showed that *C. butyricum* Sx-01, *L. salivarius* C-1-3 and their combined use did not change the blood routine indices, which indicated that *C. butyricum* Sx-01, *L. salivarius* C-1-3 and their combined use had no adverse effects on blood of mice.

### 3.3. Serum Index

As shown in Table 3, compared with the control group, the activity of total SOD in serum in group B was significantly increased (*p* < 0.05), and the activities of the total SOD, GSH-Px, and the contents of GSH in the serum in group C were significantly increased (*p* < 0.05); however, there was no significant difference between these two test groups (*p* > 0.05). Compared with the control group, group B and group C, the activities of the total SOD, GSH-Px, GSH-ST, and the amount of GSH in the serum in group D were higher than these in the other three groups (*p* < 0.05). Compared with the control group, the amounts of TC and TG in group D were lower than that in Group A (control group). These results showed that *C. butyricum*, *L. salivarius* and their combined use did not damage the liver, and the combined use of the two strains could significantly improve the antioxidant capacity and reduced the contents of TC and TG of mice.

### 3.4. VFAs

As shown in Table 4, Table 5, Table 6 and Table 7, the concentration of the acetic acid, propanoic acid, butyric acid, and total VFAs in the four groups before intragastric administration were not significantly different (*p* > 0.05). When the mice were treated, by intragastric administration, with *L. salivarius* Sx-01, *C. butyricum* C-1-3 and their mixture respectively, the butyric acid and the total VFAs were all increased on days 5, 9, and 14, and compared with the control group, the butyric acid levels in groups C and D were significantly increased (*p* < 0.05). These results showed that the combined use of the two strains could increase the butyric acid levels and the total VFAs.

### 3.5. Effect on Intestinal Morphological Structure

**Figure 1:**
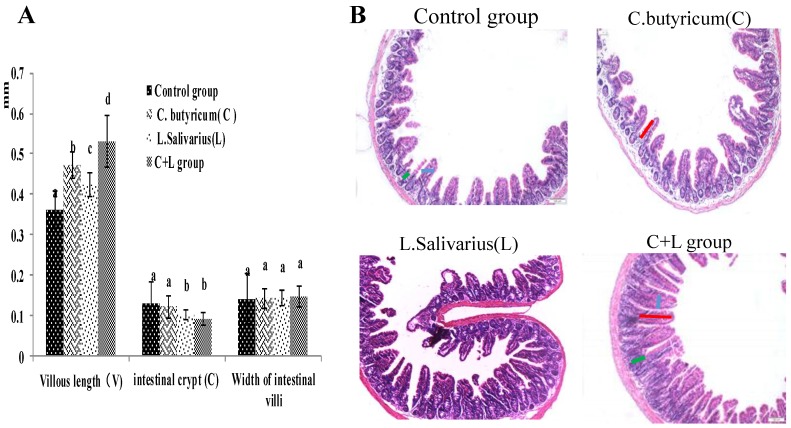
The villous length, intestinal crypt, width of intestinal villi and the ileum slice (HE 10 × 10) in different groups. (**A**) The villous length, intestinal crypt, width of intestinal villi; the ileum slice (**B**) the ileum slice (HE 10 × 10) in control group, *L. salivarius* group, *C. butyricum* group and combined treatment group. Red line represents villous length, green line represents intestinal crypt depth, blue line represents villi width. Note: On the column of different letters differed significantly (*p* < 0.05), without superscript or marked with the same letter angle has no significant difference (*p* > 0.05).

As shown in Figure 1A, compared with the control group, the villous length of the ileum was significantly increased in the *L. salivarius* group, *C. butyricum* group and with treatment by the combination thereof. The villous length was increased more significantly (*p* < 0.05) in the combined treatment group, the intestinal crypt was decreased in the *L. salivarius* group and in the combinedtreatment group (*p* < 0.05); however, the widths of the intestinal villi were not significantly different in these groups (*p* > 0.05). When the mice were treated by intragastric administration of *L. salivarius*, *C. butyricum*, or a combination thereof, the ratio of VH/CD was significantly higher (*p* < 0.05). As shown in Figure 1B, the number of villi in the combined treatment group was significantly increased, and the arrangement was more orderly than that in the control group. These results showed that the combined use of the two strains could enhance the intestine health.

**Figure 2:**
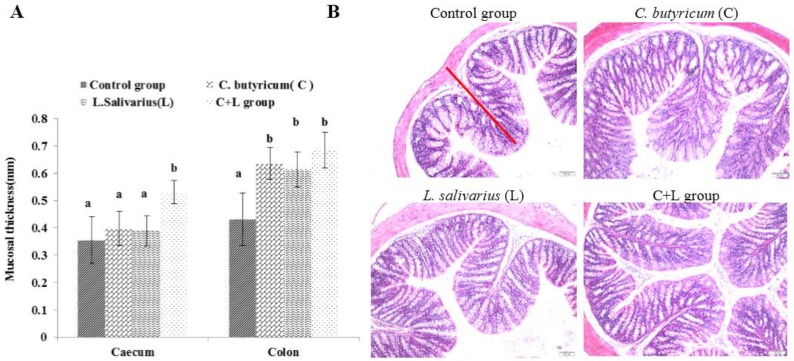
The mucosal thickness of caecum and colon and the colon slice (HE 10 × 10) slice (HE 10 × 10) in different groups. (**A**) The mucosal thicknessm; (**B**) the ileum slice (HE 10 × 10) in control group, *L. salivarius* group, *C. butyricum* group and combined treatment group. Red line represents mucosal thickness. Note: On the column of different letters differed significantly (*p* < 0.05), without superscript or marked with the same letter angle has no significant difference (*p* > 0.05).

As shown in Figure 2A, the mucosal thickness of the caecum in the combined treatment group was significantly higher than that in the control group, *L. salivarius* group, and *C. butyricum* group (*p* < 0.05). Compared with the control group, the mucosal thickness of the colon in the *L. salivarius* group, *C. butyricum* group, and the combined treatment group was increased (*p* < 0.05). As shown in Figure 2B, the mucosal thickness of the colon in the combined treatment group was significantly increased, and the arrangement was more orderly than that in the control group.

### 3.6. Effects of L. salivarius, C. butyricum, and Their Combined, Use on the Colon Microbiota

**Figure 3:**
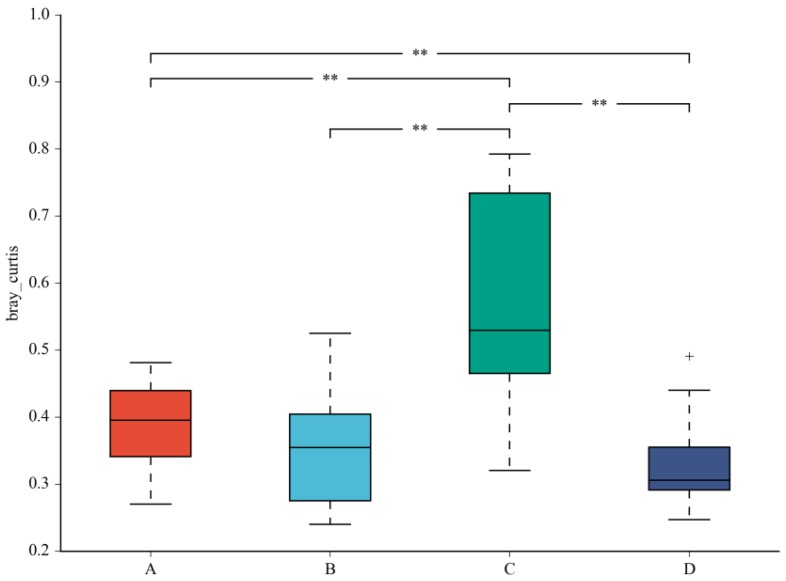
Beta diversity map based on the Bray–Curtis algorithm. Abscissa indicates group; ordinate indicates distance; boxes of different colors represent groups. ** Significant difference between groups (*p* < 0.01).The taxonomic biomarkers (LDA score > 2) in the colon microbial communities of the four groups were identified by LEfSe method (Figure 4A,B). As shown in Figure 4A,B, LEfSe detected a marked increase (LDA score *>* 2) in the relative abundance of the *Eubacterium_coprostanoligenes*_group genus and *Peptococcaceae* family in the mice of the combined treatment group compared with other groups. “+” stand for outlier.

A beta diversity map based on the Bray–Curtis algorithm (Figure 3) showed that the similarity in species diversity is very different when two strains are given to mice. The results indicated that *C. butyricum* and its combined use with *L. salivarius* could change the species diversity of bacteria in the colon of mice.

**Figure 5:**
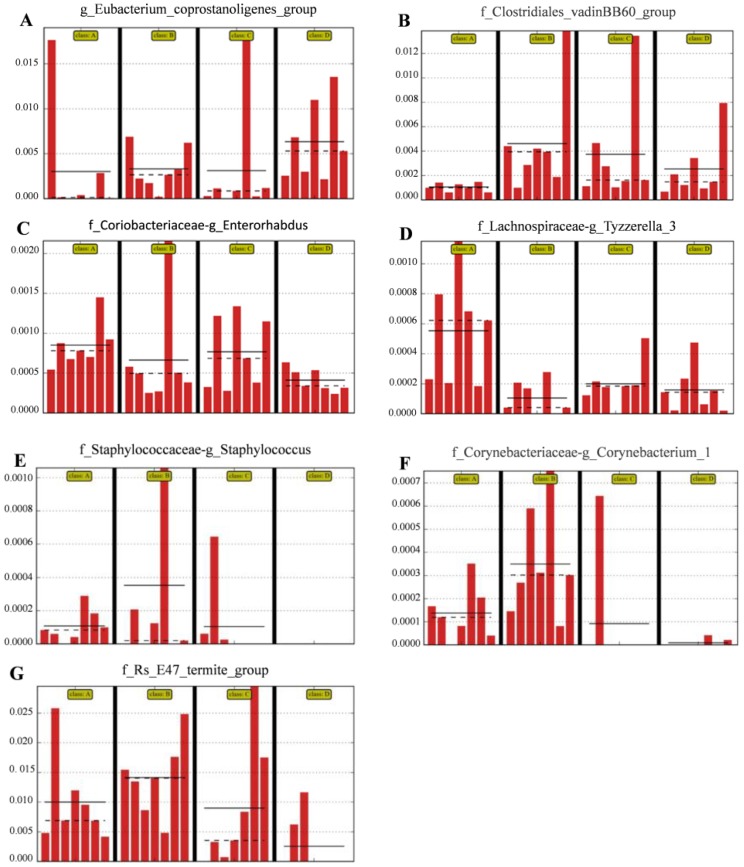
Compared with control group, the histograms indicate the higher relative abundance of the genus *Eubacterium_coprostanoligenes*_group (**A**) and family Clostridiales_vadinBB60_group (**B**) and the lower relative abundance of the genus *Enterorhabdus* (**C**), *Staphylococcus* (**E**), *Corynebacterium_1* (**F**) and *Bacteroidales-f_Rs_E47_*termite_group (**G**) in the colon microbiota of the group D. (**A**) Control group; (**B**) *L. salivarius* group; (**C**) *C. butyricum* group; (**D**) Combined treatment group.

The relative abundances of the genus *Eubacterium_coprostanoligenes*_group (Figure 5A) and family *Clostridiales*_vadinBB60_group (Figure 5B) were significantly (LDA score *>* 2) higher in group D than in the control group; however, the relative abundance of the genus *Enterorhabdus* (Figure 5C) and Tyzzerella (Figure 5D) was significantly (LDA score *>* 2) lower in group D than in the other groups; the relative abundance of *Staphylococcus* (Figure 5E), *Corynebacterium* (Figure 5F) and *Bacteroidales*-f__Rs_E47_termite_group (Figure 5G) was (LDA score *>* 2) lower in group D than that in group A (the control group).

## 4. Discussion

Previous studies showed that combined administration of probiotic stains of *B. subtilis* and *L. acidophilus* to weaning rabbits could improve the growth performance, cecal fermentation and number of gut beneficial bacteria populations [27]; the supplementation of *B. subtilis*, *C. butyricum* and *L. acidophilus* together improved growth performance, nutrient digestibility, and gut health in broilers [28]; the supplementation of Bacillus, Saccharomyces and lactic acid bacteria (LAB) had positive effects on growth, feed conversion and nutrient digestibility in grower pigs and increased faecal LAB counts and decreased faecal *E. coli* counts in the grower pigs [29]. These results indicated that combined use of probiotic could increase animal production and to a certain extent, the probiotic effects were related to the regulation of gut microbiota. However, there is no report on the prebiotic effect of combining the use of strains of *C. butyricum* and and *L. salivarius*.

In our study, we firstly confirmed the safety of *C. butyricum* Sx-01 and *L. salivarius* C-1-3 from the results of blood biochemical and then found that the combined use of the two strains could improve intestinal health and reduce blood lipids, which might be related to their regulating intestinal flora besides their own probiotic effects.

In our tests, the mice were given 4 × 10^8^ cfu of the two strains per day. The dosage was close to most experiments in mice [2,12,16,30,31]. The safety of *C. butyricum* was vitally important. In our tests, *C. butyricum* Sx-01 did not show any toxicological effects in mice as there were no adverse effects on the blood and biochemical indices at the oral dosage with 4 × 10^8^ cfu to the mice. Furthermore, the *C. butyricum* Sx-01 strain was separated from the healthy and untreated chicken intestines, while toxigenic strains of *C. butyricum* were often found in pathological conditions, such as botulism in infants or necrotizing enterocolitis in neonates [13,32,33,34]. Until now, *L. salivarius* does not pose a health risks to animals or humans in the doses currently used for a variety of applications [15]. Our results also showed that *L. salivarius* C-1-3 was safe to use as it had no adverse effects on the blood and biochemical indices and could improve intestinal health. Combined with our previous study, *L. salivarius* C-1-3 had the characters of the acid, bile and high temperature resistance and had a powerful antibacterial effect against *E. coli*, *Salmonella* and *Staphylococcus aureus* [20]; it could be considered as a probiotic.

In recent years, some studies showed that combined use of probiotics could promote growth performance in animals [35,36,37,38]. However, some research showed that combined use of probiotics exerted no significant effect on growth performance [39]. Our results found that compared with control, the effect of *C. butyricum* Sx-01, *L. salivarius* C-1-3, and their combined use on the growth performance of mice was not significant, but the average daily gain and feed utilisation showed an increasing trend. The inconsistency might be related to the strains of probiotic, administration dosage, diet composition, the experimental duration and experimental animals. Therefore, in the next step, the experimental design should be extended to give other animals different dosages and observe any longer-term effects.

Our results showed that the average haemoglobin concentration in the serum of mice increased when the mice were given *C. butyricum* Sx-01, or *L. salivarius* C-1-3. These results might be that these two probiotics enhance the circulation of oxygen in mice and enhance the metabolic function of mice. However, the mechanisms need to be further studied. 

Studies have shown that probiotics can enhance antioxidant ability [40,41]. Consistent with our results, the findings showed that serum antioxidant abilities were significantly enhanced while using *L. plantarum* [42], *L. johnsonii* BS15 [43] and yeast [44]. Our results showed that when mice were given *C. butyricum* and *L. salivarius*, the activities of SOD, GSH-Px in the serum were increased, and the MDA content was decreased. SOD and GSH-Px are important enzymes in the defence against oxidative stress and MDA is the main product of polyunsaturated lipid peroxidation. Previous studies have demonstrated that some strains of *C. butyricum* and *L. salivarius* can decrease the oxidative state by enhancing the levels of the activities of SOD and GSH-Px and decreasing the level of MDA to reduce the production of reactive oxygen species (ROS) [45,46]. The mechanism of antioxidation may be that the SOD and GSH produced by *Lactobacillus* can scavenge hydroxyl radicals and hydrogen peroxide [47] and butyrate and H_2_ produced by *C. butyricum* have beneficial effects on reduction of oxidative stress [48]. Our results further proved that the two probiotics could improve the antioxidant capacity and also demonstrated that improving the antioxidant capacity by combining the use of the two strains was better than their single use. The reason could be that when combining use of the two strains, the antioxidant capacity was superimposed.

Our results showed that upon administration of mice with the mixture of *C. butyricum* Sx-01 and *L. salivarius* C-1-3, the triglyceride, and glycaemic, indices were significantly reduced in the serum compared with those in the control, and single-use groups. One reason for this might be that some strains of *Lactobacillus* had beneficial effects on glycaemic control, triglycerides, and VLDL cholesterol concentrations [49,50] and *C. butyricum* could decrease plasma and hepatic cholesterol levels in cholesterol-fed rats [10]. When the two species were administrated together to the mice, they could play a synergistic role in retarding cholesterol synthesis or increasing degradation of cholesterol by gut bacterial enzymes [45,51]. Interestingly, we found that the two probiotics, in combined use, enhanced the relative abundance of the genus *Eubacterium_coprostanoligenes*_group, by which cholesterol could be decomposed into faecal sterols that were not absorbed but were excreted in faeces [52,53,54].

Villus height and crypt depth are reliable indicators of gut function and health. The increase of intestinal villus length can strengthen the contact between the intestine and nutrients and improve digestion and absorption. Previous studies showed that dietary supplementation with *C. butyricum* benefited ideal morphology in broilers [55] and dietary inclusion of a microbial feed additive (*L. salivarius* and *L. reuteri*) improved intestinal nutrient absorption which was in association with an improvement in intestinal architecture [56]. Our results showed that the intestinal mucosa, in all test groups, remained intact, the structure of each layer was clear, and the order of intestinal villi was regular. Furthermore, the effect of the two strains applied as a mixture on the length of the intestinal villi was much better than that conferred by their separate administration. These results demonstrated that the combination of the two probiotics could better affect the development of intestinal health and promote the digestion and absorption function of mice. The main reason might be that the combination of the two probiotics decreased the relative abundance of some pathogenic bacteria of the genus *Enterorhabdus*, *Staphylococcus* and *Corynebacterium* in the colon (Figure 5E,H,I), which can damage the intestinal mucosal epithelial cells (IECs). The other reason might be that the butyric acid, which is beneficial for IECs [57,58], was increased upon intragastric administration of *C. butyricum* (to produce butyric acid) (Table 7), and when the combined use of the two bacteria contribute to improve the family *Clostridiales*_vadinBB60_group (also producing butyric acid) (Figure 5C).

*Enterorhabdus* spp. was found in the ileitis and colitis of mice and in the human gut, which was related to inflammatory diseases [59,60]. *Staphylococcus* was associated with allergy development in the gut [61]. Some strains of *Corynebacterium* could induce genitourinary infection and caused a chronic contagious infectious disease of the caseous lymphadenitis or pseudotuberculosis [62,63]. We found that the abundances of *Enterorhabdus* spp., *Staphylococcus* spp. and *Corynebacterium* spp. in the colon were significantly reduced when administration the mice with the mixture of the two strains. These results further confirm that the combination use of the two strains enhances intestinal health via regulating the intestinal microflora and inhibiting harmful bacteria in the intestine. 

Interestingly, we found that when the two probiotics were used together, the abundance of Tyzzerella was significantly reduced. A previous study showed that *Tyzzerella* was correlated with risk of cardiovascular disease [64]. Our results indicated that the combined use of the two bacteria might reduce the risk of cardiovascular disease, however, whether the simultaneous use of the two probiotics could actually reduce cardiovascular risk needs further investigation. 

## 5. Conclusions

The combined use of *C. butyricum* Sx-01 and *L. salivarius* C-1-3 could significantly improve intestinal health, improve the antioxidant capacity of the body, and reduce the amount of lipids in sera of mice. The reason for this might be that besides their own probiotic effects, combined administration of the two strains was beneficial in that it increased the number of healthy intestinal microflora and decreased the number of harmful intestinal microflora.

## Figures and Tables

**Figure 4:**
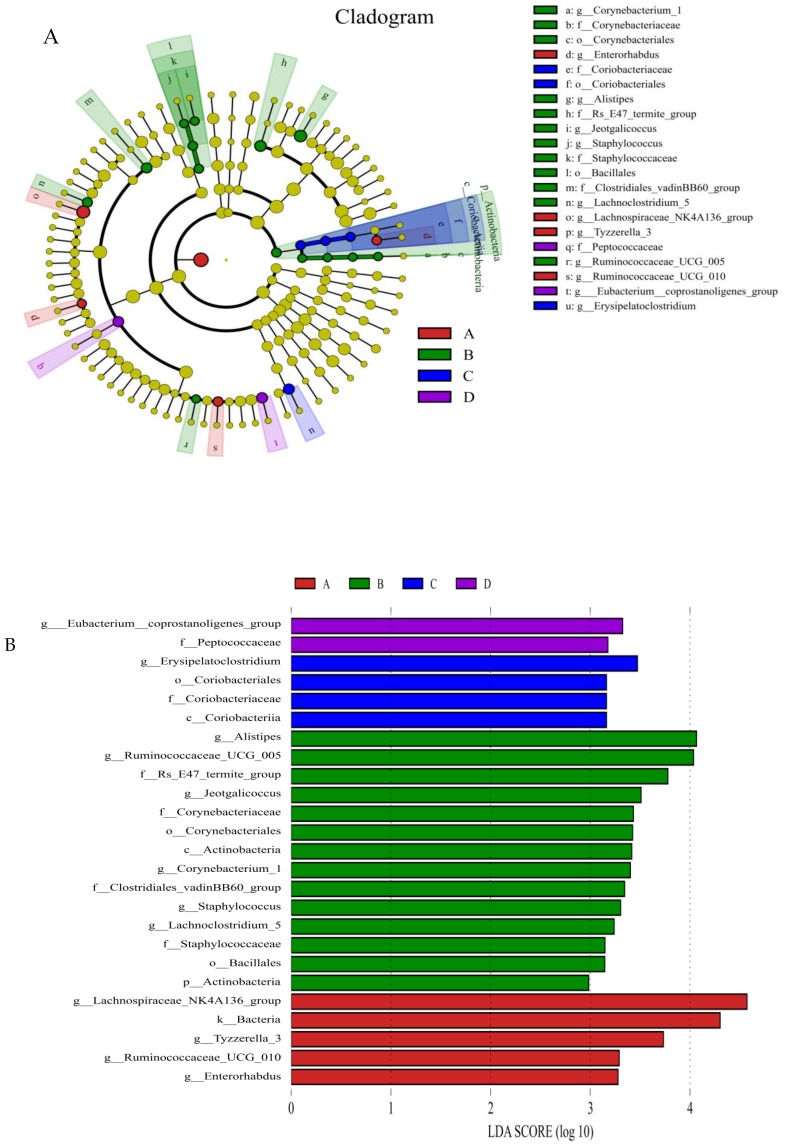
Diversity and composition of colon microbiota. LEfSe results in the four groups, representing the relevant features on taxonomic trees (**A**) and Key phylotypes in the colon responding to treatments were identified by the LEfSe algorithm (**B**). (**A**) Control group; (**B**) *L. salivarius* group; (**C**) *C. butyricum* group; (**D**) Combined treatment group.

**Table 1:** Effect of intragastric administration with *C. butyricum* Sx-01, *L. salivarius* C-1-3 and a combination thereof on growth performance of mice.

Index	Group A	Group B	Group C	Group D
average daily gain (g)	0.71 ± 0.07	0.75 ± 0.08	0.76 ± 0.09	0.74 ± 0.10
average daily feed intake (g)	4.77 ± 0.53	4.82 ± 0.09	4.47 ± 0.38	4.77 ± 0.29
Feed/Gain Ratio (%)	6.73 ± 1.13	6.49 ± 0.69	5.96 ± 1.05	6.51 ± 0.69
diarrhea rate (%)	0	0	0	0
mortality (%)	0	0	0	0

**Table 2:** Effect of intragastric administration with *C. butyricum* Sx-01, *L. salivarius* C-1-3 and a combination thereof on blood indices of mice.

Index	Group A	Group B	Group C	Group D
WBC (×10^9^/L)	4.24 ± 2.04	3.80 ± 1.93	3.96 ± 0.99	2.78 ± 1.55
LY (%)	60.42 ± 6.25	51.25 ± 10.27	56.74 ± 8.02	61.16 ± 8.23
MID (%)	10.70 ± 1.15	10.03 ± 2.61	9.08 ± 5.85	13.64 ± 2.46
GR (%)	28.88 ± 5.55 ^ab^	38.73 ± 12.35 ^a^	32.38 ± 7.87 ^ab^	25.20 ± 6.03 ^b^
LY (×10^9^/L)	2.48 ± 1.04	1.98 ± 1.05	2.24 ± 0.66	1.24 ± 1.04
MID (×10^9^/L)	0.48 ± 0.27	0.38 ± 0.21	0.42 ± 0.15	0.38 ± 0.22
GR(×10^9^/L)	1.28 ± 0.80	1.45 ± 0.84	1.20 ± 0.51	0.76 ± 0.47
RBC (×10^12^/L)	8.03 ± 1.22	7.29 ± 2.68	7.74 ± 2.28	7.75 ± 2.93
HGB (g/L)	129.80 ± 24.31	126.25 ± 33.94	122.00 ± 34.95	121.80 ± 42.77
HCT (%)	44.13 ± 6.32	36.57 ± 14.00	37.12 ± 10.15	36.72 ± 13.71
MCV (fL)	55.13 ± 2.24 ^a^	49.90 ± 1.57 ^b^	48.48 ± 2.83 ^b^	47.70 ± 2.70 ^b^
MCH (Pg)	15.10 ± 0.80	16.30 ± 1.49	15.76 ± 0.25	15.98 ± 1.09
MCHC (g/L)	275.25 ± 23.47 ^b^	328.67 ± 40.20 ^a^	327.40 ± 15.95 ^a^	336.40 ± 18.90 ^a^
RDW-CV (%)	18.93 ± 4.50	16.63 ± 1.56	16.26 ± 1.42	14.68 ± 4.24
RDW-SD (fL)	45.10 ± 7.43 ^a^	34.67 ± 2.71 ^b^	32.50 ± 0.92 ^b^	33.02 ± 1.48 ^b^
PLT (×10^9^/L)	426.80 ± 143.29	281.33 ± 86.01	310.20 ± 123.43	338.80 ± 104.37
MPV (fL)	8.24 ± 0.76	7.63 ± 0.32	8.18 ± 1.90	7.42 ± 0.35
PDW (fL)	10.95 ± 3.32	9.30 ± 1.56	8.00 ± 2.71	7.78 ± 1.52
PCT (%)	0.34 ± 0.13	0.21 ± 0.08	0.26 ± 0.13	0.25 ± 0.08

*Note: WBC, white blood cell; LY, lymphocytes; MID, median cell; GR, granulocyte; RBC, red blood cell; HGB, Hemoglobin; HCT, hematocrit; MCV, mean corpusular hemoglobin; MCH, mean corpusular hemoglobin; MCHC, mean corpusular hemoglobin concentration; RDW-CV, red blood cell volume distribution width-Coefficient of Variation; RDW-SD, red blood cell volume distribution width-standard deviation; PLT, blood platelet; MPV, Meanplateletvolume; PDW, Platelet distribution width; PCT, thrombocytocrit.* On the corner of different letters differed significantly (*p* < 0.05), without superscript or marked with the same letter angle has no significant difference (*p* > 0.05).

**Table 3:** Effect of intragastric administration with *C. butyricum* Sx-01, *L. salivarius* C-1-3 and a combination thereof on blood indices of mice.

Index	Group A	Group B	Group C	Group D
T-SOD (U/mL)	99.276 ± 5.752 ^b^	104.649 ± 4.444 ^a^	107.227 ± 5.128 ^a^	110.336 ± 5.150 ^c^
GSH-ST (U/mL)	17.521 ± 0.676 ^a^	17.982 ± 2.457 ^a^	18.990 ± 1.525 ^a^	20.978 ± 1.642 ^b^
GSH-Px (U/mL)	663.334 ± 74.437 ^b^	678.222 ± 77.299 ^ab^	734.222 ± 65.212 ^a^	749.618 ± 70.621 ^c^
GSH (umol/L)	24.568 ± 1.985 ^b^	25.828 ± 3.079 ^ab^	27.635 ± 3.473 ^a^	29.898 ± 2.275 ^c^
MDA (nmol/mL)	6.085 ± 1.389 ^a^	5.662 ± 1.257 ^ab^	4.763 ± 1.193 ^b^	4.754 ± 1.291 ^b^
ALB (g/L)	16.850 ± 1.228 ^ab^	17.290 ± 1.073 ^a^	16.018 ± 0.947 ^ab^	15.960 ± 1.836 ^b^
TP (g/L)	31.263 ± 2.605	32.180 ± 2.623	30.336 ± 1.938	30.970 ± 2.394
GLO (g/L)	14.413 ± 1.547	14.890 ± 1.838	14.318 ± 1.160	15.010 ± 1.323
AST (U/L)	59.938 ± 14.961	55.720 ± 5.466	54.936 ± 10.593	60.880 ± 12.438
ALT (U/L)	25.325 ± 7.117 ^a^	22.060 ± 3.858 ^ab^	21.364 ± 8.026 ^a^	19.210 ± 2.937 ^b^
TC (mmol/L)	1.361 ± 0.071 ^a^	1.394 ± 0.334 ^a^	1.285 ± 0.206 ^a^	1.071 ± 0.033 ^b^
TG (mmol/L)	1.198 ± 0.371 ^a^	0.956 ± 0.224 ^a^	1.189 ± 0.263 ^a^	0.861 ± 0.280 ^b^

Note: On the corner of different letters differed significantly (*p* < 0.05), without superscript or marked with the same letter angle has no significant difference (*p* > 0.05).

**Table 4:** Effect of intragastric administration with *C. butyricum* Sx-01, *L. salivarius* C-1-3 and a combination thereof on blood excrement Volatile fatty acids (VFAs) concentration of mice (Day 0).

	Acetic Acid	Propanoic Acid	Butyric Acid	Total VFAs
Group A	53.04 ± 7.24	11.17 ± 1.27	4.92 ± 0.27	69.13 ± 8.61
Group B	48.08 ± 1.84	10.36 ± 1.32	5.01 ± 1.74	63.45 ± 3.92
Group C	47.86 ± 4.19	11.74 ± 1.56	4.99 ± 1.09	64.59 ± 5.80
Group D	47.27 ± 3.57	9.13 ± 0.60	6.68 ± 1.25	63.08 ± 5.41

**Table 5:** Effect of intragastric administration with *C. butyricum* Sx-01, *L. salivarius* C-1-3 and a combination thereof on blood excrement VFAs concentration of mice (Day 5).

	Acetic Acid	Propanoic Acid	Butyric Acid	Total VFAs
Group A	47.88 ± 1.45	9.76 ± 0.14 ^b^	4.60 ± 0.44 ^c^	62.25 ± 1.88
Group B	49.95 ± 5.06	11.27 ± 1.68 ^ab^	4.76 ± 0.58 ^c^	65.98 ± 7.24
Group C	52.00 ± 5.48	11.99 ± 1.08 ^a^	7.75 ± 0.49 ^a^	71.74 ± 7.05
Group D	50.36 ± 1.83	10.55 ± 0.65 ^ab^	7.70 ± 0.43 ^a^	68.61 ± 1.30

Note: On the corner of different letters differed significantly (*p* < 0.05) without superscript or marked with the same letter angle has no significant difference (*p* > 0.05).

**Table 6:** Effect of intragastric administration with *C. butyricum* Sx-01, *L. salivarius* C-1-3 and a combination thereof on blood excrement VFAs concentration of mice (Day 9).

	Acetic Acid	Propanoic Acid	Butyric Acid	Total VFAs
Group A	49.12 ± 0.19 ^b^	9.46 ± 0.22 ^c^	4.99 ± 0.90 ^b^	63.57 ± 1.18 ^b^
Group B	48.31 ± 3.17 ^b^	10.52 ± 0.94 ^bc^	5.46 ± 1.42 ^b^	64.28 ± 5.37 ^b^
Group C	65.22 ± 6.43 ^a^	15.00 ± 3.24 ^a^	8.14 ± 1.85 ^a^	88.36 ± 11.53 ^a^
Group D	65.26 ± 3.08 ^a^	13.55 ± 1.07 ^ab^	8.70 ± 0.75 ^a^	87.52 ± 4.20 ^a^

Note: On the corner of different letters differed significantly (*p* < 0.05) without superscript or marked with the same letter angle has no significant difference (*p* > 0.05).

**Table 7:** Effect of intragastric administration with *C. butyricum* Sx-01, *L. salivarius* C-1-3 and a combination thereof on blood excrement VFAs concentration of mice (Day 14).

	Acetic Acid	Propanoic Acid	Butyric Acid	Total VFAs
Group A	73.77 ± 25.84	12.44 ± 4.48	8.35 ± 0.27 ^b^	88.56 ± 30.21 ^b^
Group B	74.33 ± 27.26	16.38 ± 3.57	12.13 ± 3.68 ^ab^	102.85 ± 34.42 ^ab^
Group C	75.63 ± 23.59	17.92 ± 5.32	14.32 ± 2.75 ^a^	107.87 ± 31.66 ^a^
Group D	76.87 ± 22.36	16.65 ± 3.95	14.39 ± 2.35 ^a^	105.81 ± 28.60 ^a^

Note: On the corner of different letters differed significantly (*p* < 0.05), without superscript or marked with the same letter angle has no significant difference (*p* > 0.05).
